# Comparison of performance in neuropsychological tests in amnestic Mild Cognitive Impairment and Alzheimer’s disease patients

**DOI:** 10.1590/S1980-57642009DN30100009

**Published:** 2009

**Authors:** Patrícia Helena Figueirêdo do Vale, Lívia Spíndola, Maira Okada de Oliveira, Cristiane Garcia da Costa Armentano, Claudia Sellitto Porto, Sonia Maria Dozzi Brucki

**Affiliations:** 1 Psychologist, Behavioral and Cognitive Neurology Unit, Department of Neurology of the University of São Paulo School of Medicine and Cognitive Disorders Reference Center (CEREDIC), Hospital das Clínicas of the University of São Paulo School of Medicine, São Paulo, SP, Brazil.; 2 PhD, Behavioral and Cognitive Neurology Unit, Department of Neurology of the University of São Paulo School of Medicine and Cognitive Disorders Reference Center (CEREDIC), Hospital das Clínicas of the University of São Paulo School of Medicine, São Paulo, SP, Brazil.; 3 MD, PhD, Behavioral and Cognitive Neurology Unit, Department of Neurology of the University of São Paulo School of Medicine and Cognitive Disorders Reference Center (CEREDIC), Hospital das Clínicas of the University of São Paulo School of Medicine, São Paulo, SP, Brazil.

**Keywords:** Mild Cognitive Impairment, Amnestic Mild Cognitive Impairment, Alzheimer’s disease, neuropsychological tests, memory

## Abstract

Mild Cognitive Impairment (MCI) can be an intermediate state between normality and dementia in some patients. An early diagnosis, through neuropsychological assessment, could identify individuals at risk of developing dementia.

**Objective:**

To verify differences in performance on neuropsychological tests among controls, amnestic MCI (aMCI) and Alzheimer’s disease (AD) patients.

**Methods:**

Sixty-eight AD patients (mean age 73.77±7.24; mean schooling 9.04±4.83; 40 women and 28 men), 34 aMCI patients (mean age 74.44±7.05; mean schooling 12.35±4.01; 20 women) and 60 controls (mean age 68.90±7.48; mean schooling 10.72±4.74; 42 women) were submitted to a neuropsychological assessment composed of tasks assessing executive functions, language, constructive abilities, reasoning and memory.

**Results:**

There were statistically significant differences in performance across all tests among control, aMCI and AD groups, and also between only controls and AD patients. On comparing control and aMCI groups, we found statistically significant differences in memory tasks, except for immediate recall of Visual Reproduction. There were also statistically significant differences between aMCI and AD groups on tasks of constructive and visuoperceptual abilities, attention, language and memory, except for delayed recall of Visual Reproduction.

**Conclusions:**

Neuropsychological assessment was able to discriminate aMCI from AD patients in almost all tests except for delayed recall of Visual Reproduction, visual organization (Hooper) and executive functions (WCST); and discriminate controls from AD patients in all tests, and controls from aMCI patients in all memory tests except for immediate recall of Visual Reproduction.

## Abstract (pt)

Comprometimento Cognitivo Leve (CCL) pode ser um estágio entre normalidade e demência em alguns pacientes. Um diagnóstico precoce, com avaliação neuropsicológica, pode identificar indivíduos com risco para desenvolvimento de demência.

**Objetivo:**

Verificar diferenças no desempenho em testes neuropsicológicos entre controles, pacientes com CCL amnéstico (CCLa) e com doença de Alzheimer (DA).

**Métodos:**

Sessenta e oito pacientes com DA (média de idade 73,77±7,24; média de escolaridade 9,04±4,83; 40 mulheres), 34 pacientes com CCLa (média de idade 74,44±7,05; média de escolaridade 12,35±4,01; 20 mulheres) e 60 controles (média de idade 68,90±7,48; média de escolaridade 10,72±4,74; 42 mulheres) foram submetidos à ampla avaliação neuropsicológica.

**Resultados:**

Houve diferenças estatisticamente significativas em todos os testes entre os grupos controle, CCLa e DA, e entre controles e o grupo DA. Na comparação entre controles e pacientes com CCLa foram encontradas diferenças estatisticamente significativas nos testes de memória, exceto na evocação imediata do teste de Reprodução Visual. Foram também encontradas diferenças estatisticamente significativas entre pacientes com CCLa e DA, nas tarefas de habilidades construtivas e visuoperceptuais, atenção, linguagem e memória, com exceção da evocação tardia do teste Reprodução Visual.

**Conclusões:**

A avaliação neuropsicológica foi capaz de discriminar pacientes com CCLa de pacientes com DA em quase todos os testes exceto na evocação tardia do teste Reprodução Visual, organização visual (Hooper) e funções executivas (WCST); controles de pacientes com DA em todos os testes, e controles de pacientes com CCLa nos testes de memória, exceto na evocação imediata do teste Reprodução Visual.

Mild Cognitive Impairment (MCI) is a term proposed by Petersen et al.^1^ to report a clinical syndrome presented by individuals with measurable cognitive deficits who do not fulfill criteria for diagnosis of dementia but who are at high risk of developing it, mainly in the form of Alzheimer’s disease (AD).^2^

The diagnosis of MCI is established based on evidence of cognitive disorder in non-demented individuals who present complaints (preferably corroborated by an informant) in conjunction with objectively measured cognitive deficits, preserved activities of daily living, and intact or minimally impaired complex instrumental functions.^2,3^

The clinical presentations of MCI can be classified accordingly to three subtypes: amnestic MCI (aMCI), multiple domains (mdMCI) and nonmemory single domain (naMCI single domain) MCI. In aMCI, memory is impaired, but the other cognitive functions can be preserved. On the other hand, mdMCI is characterized by cognitive impairments in other domains such as language, attention, executive functions or visuospatial skills. In these cases, if memory is significantly impaired then this confers aMCI multiple domains, and if not naMCI multiple domains. In the naMCI single domain only one function beyond memory is impaired (e.g. language or visuospatial).^2,3^

Subjects with MCI can progress to AD or other dementia, remain stable or even recover, although when a person with aMCI has a suspected degenerative disorder this is most likely a case of prodromal AD.^2-4^

Data on the prevalence of MCI and the conversion rate to dementia vary widely, depending on the different defining criteria applied^5^. In a cohort study, Fisher et al.^6^ compared the conversion rate of the subtypes of MCI to AD in elderly subjects, followed for 30 months. The conversion rates were 48.7% for subjects with aMCI and 28.6% for those with non-amnestic MCI. The rate of conversion to AD among healthy subjects was 12.6%. Visser et al.^7^ conducted a 10-year follow up study involving subjects with cognitive impairment aged 40 to 85 years to investigate the risk of dementia, and reported rates of conversion of 27% in subjects with cognitive complaints, 28% in subjects with aging-associated cognitive decline, 44% in subjects with mild functional impairment, and 48% in subjects with amnestic MCI.

Controversy exists as to how MCI can be best assessed, as there is insufficient evidence to recommend specific tests^2^. Petersen et al.^1^ compared MCI patients, mild AD and controls on a neuropsychological battery and observed that subjects with MCI performed worse than controls, but better than AD patients on memory tasks, and proved more similar to control subjects than to AD patients on measures of general cognition and other nonmemory indexes. In longitudinal studies, delayed recall tests were found to be good predictors of progression to AD^8,9^, while poor performance in delayed recall and executive function tests indicate a high risk of progression to dementia. Arnaiz and Almkvist^4^ believe that beyond memory tests, tasks evaluating other cognitive functions such as verbal abilities and learning, visuospatial function, attention and executive functions are important for screening and diagnosis of MCI and early AD.

The aim of this study was to verify differences in performance on neuropsychological tests by controls, aMCI and AD patients.

## Methods

In this retrospective study we reviewed 102 medical files of subjects which were divided into three groups: controls (n=60), Alzheimer’s disease patients (n=68), and aMCI patients (n=34) (Table 1).

**Table 1:** Demographics of the sample.

	Controls (n=60)			aMCI (n=34)			AD (n=68)		*p*
	Mean	SD		Mean	SD		Mean	SD	
Gender (female/male)	42/18			20/14			40/28		0.364*
Age	68.90	±7.48		74.44	±7.05		73.77	±7.24	<0.001**
Education	10.72	±4.74		12.35	±4.01		9.04	±4.83	0.03**

* Chi-Square Test;

** Kruskall-Wallis Test; SD, standard deviation.

All patients were evaluated by members of the Behavioral and Cognitive Neurology Unit of the Department of Neurology at the University of Sao Paulo School of Medicine and from the Center of Cognitive Disorders (CEREDIC) and had been submitted to a general clinical and neurological evaluation and to screening tests comprising the Mini-Mental State Examination (MMSE)^10^, Brief Cognitive Screening Battery (BCSB),^11,12^ and Functional Activities Questionnaire.^13,14^

The patients were classified with mild dementia according to the American Psychiatric Association, Diagnostic and Statistical Manual of Mental Disorders, Third Edition, revised (DSM-III-R)^15^ criteria and probable AD according to National Institute of Neurological Disorders and Communicative Disorders and Stroke Alzheimer Disease and Related Disorders Association (NINCDS-ADRDA) criteria^16^. Amnestic MCI patients were diagnosed based on the criteria proposed by Petersen et al.^3,17^

All patients were also submitted to a comprehensive neuropsychological battery of tests including: executive functions (Trail Making Test versions A and B, Wisconsin Card Sorting Test – WCST);^18^ visual perception (Hooper Visual Organization Test,^19^ Raven’s Progressive Matrices^20^); language (Boston Naming Test,^21,22^ semantic verbal fluency^18^); constructive abilities (Block Design, Wechsler Adult Intelligence Scale – WAIS^23^, Rey Complex Figure Copy^18,24^); visual and verbal memory tests (Visual Reproduction of the Wechsler Memory Scale – Revised - WMS-R,^25^ Rey Complex Figure – delayed recall^18,24^, WMS-R Logical Memory,^25^ Rey Auditory Verbal Learning Test – RAVLT^26^).

Cases of moderate or severe AD, and those presenting other disorders that could affect cognitive functions and non-corrected visual or auditory disorders were excluded.

The control group was composed of 60 volunteers (42 women and 18 men) with no memory or functional disturbances. To be included they had to score above the median values for their educational level on the MMSE,^10^ below 22 in the Memory Complaint Questionnaire (MAQ-Q)^27^ and below 3.84 on the Informant Questionnaire on Cognitive Decline in the Elderly (IQCODE).^28^ Subjects with neurological disease, history of alcoholism, depression, or any other psychiatric disorder, non-corrected visual or auditory disorders, motor disorders, or users of psychotropic drugs that could affect cognitive functions were excluded. Chronic diseases such as hypertension, diabetes mellitus and cardiac disorders, if adequately controlled, were not criteria for exclusion.

All patients and control individuals who agreed to participate in the study signed a written informed consent term. This study was approved by the Ethics Committee of the Hospital das Clínicas of the University of São Paulo School of Medicine.

### Statistical analysis

We used the Chi-square test to evaluate associations between the categorical variables and the results. When the variables were continuous, the comparisons were performed by Mann-Whitney for two samples, and by the Kruskall-Wallis test for more than two samples. The value of significance accepted was 0.01.

All statistical analysis was carried out using the program *Statistical Package for the Social Sciences (SPSS)*, for *Windows*, version 10.0.

## Results

No differences related to gender (*p=*0.364) were found among the control group, aMCI and AD patients, but statistically significant differences related to age (*p*<0.001) and schooling (*p=*0.03) were observed (Table 1).

Comparison among controls, aMCI and AD patients on neuropsychological assessments yielded statistically significant differences in all tests applied (Table 2).

**Table 2:** Performance of controls, aMCI and AD patients on neuropsychological tests*.

Tests	Mean±SD			*p*
	Controls	MCI	AD	
Hooper	54.73 (14.87)	55.60 (17.54)	64.02 (19.75)	0.007
Block Design (WAIS)	10.50 (2.75)	11.66 (2.50)	7.10 (3.74)	<0.001
Rey Figure - Copy	30.64 (6.86)	28.20 (6.60)	21.39 (10.18)	<0.001
Rey Figure - Memory	11.02 (7.69)	2.51 (3.21)	0.79 (2.69)	<0.001
Trail Making - Part A (Time)	50.81 (31.99)	47.53 (15.23)	92.76 (46.49)	<0.001
Trail Making - Part B (Time)	113.72 (52.32)	110.25 (41.75)	215.20 (83.89)	<0.001
Logical Memory (WMS-R) - immediate	33.17 (5.51)	26.03 (8.14)	13.78 (8.84)	<0.001
Logical Memory (WMS-R) - 30'	16.94 (8.53)	4.66 (5.33)	1.35 (2.93)	<0.001
Visual Reproduction (WMS-R) - immediate	28.82 (8.12)	22.66 (7.57)	14.20 (7.33)	<0.001
Visual Reproduction (WMS-R) - 30'	13.64 (11.81)	1.23 (4.32)	1.01 (3.95)	<0.001
RAVLT - total	40.08 (8.19)	28.60 (7.81)	21.00 (7.91)	<0.001
RAVLT - 30'	7.55 (3.24)	2.31 (1.74)	1.06 (2.82)	<0.001
BNT	45.02 (6.85)	42.71 (6.22)	34.28 (8.93)	<0.001
WCST (Categories)	2.12 (1.54)	1.40 (1.34)	0.43 (0.62)	0.001
Verbal Fluency - supermarket	20.81 (3.74)	18.80 (5.82)	13.49 (4.74)	<0.001
Raven's Colored Matrices	26.90 (4.70)	26.50 (3.50)	17.44 (6.22)	<0.001

* Kruskall-Wallis Test; SD, standard deviation; MCI, Mild Cognitive Impairments; WAIS, Wechsler Adult Intelligence Scale; WMS-R, Wechsler Memory Scale - Revised; RAVLT, Rey Auditory Verbal Learning Test; BNT, Boston Naming Test; WCST, Wisconsin Card Sorting Test.

When control group and AD patients were compared, statistically significant differences were found in all tests (Table 3).

**Table 3:** Performance of controls and AD patients on the neuropsychological tests*.

Tests	Mean±SD		*p*
	Controls	AD	
Hooper	54.73 (14.87)	64.02 (19.75)	0.004
Block Design (WAIS)	10.50 (2.75)	7.10 (3.74)	<0.001
Rey Figure - Copy	30.64 (6.86)	21.39 (10.18)	<0.001
Rey Figure - Memory	11.02 (7.69)	0.79 (2.69)	<0.001
Trail Making - Part A (Time)	50.81 (31.99)	92.76 (46.49)	<0.001
Trail Making - Part B (Time)	113.72 (52.32)	215.20 (83.89)	<0.001
Logical Memory (WMS-R) - immediate	33.17 (5.51)	13.78 (8.84)	<0.001
Logical Memory (WMS-R) - 30'	16.94 (8.53)	1.35 (2.93)	<0.001
Visual Reproduction (WMS-R) - immediate	28.82 (8.18)	14.20 (7.33)	<0.001
Visual Reproduction (WMS-R) - 30'	13.64 (11.81)	1.01 (3.95)	<0.001
RAVLT - total	40.08 (8.19)	21.00 (7.91)	<0.001
RAVLT - 30'	7.55 (3.24)	1.06 (2.82)	<0.001
BNT	45.02 (6.85)	34.28 (8.93)	<0.001
WCST (Categories)	2.12 (1.54)	0.43 (0.62)	<0.001
Verbal Fluency - supermarket	20.81 (3.74)	13.49 (4.74)	<0.001
Raven's Colored Matrices	26.90 (4.70)	17.44 (6.22)	<0.001

* Mann-Whitney Test; SD, standard deviation; MCI, Mild Cognitive Impairments; WAIS, Wechsler Adult Intelligence Scale; WMS-R, Wechsler Memory Scale - Revised; RAVLT, Rey Auditory Verbal Learning Test; BNT, Boston Naming Test; WCST, Wisconsin Card Sorting Test.

Comparison between the control group and aMCI patients revealed statistically significant differences in memory tasks (visual and verbal) for immediate and delayed recall, except for the immediate recall of Visual Reproduction test (Table 4).

**Table 4:** Performance of controls and aMCI patients on the neuropsychological tests*.

Tests	Mean±SD		*p*
	Controls	MCI	
Hooper	54.73 (14.87)	55.60 (17.54)	0.280
Block Design (WAIS)	10.50 (2.75)	11.66 (2.50)	0.074
Rey Figure - Copy	30.64 (6.86)	28.20 (6.60)	0.079
Rey Figure - Memory	11.02 (7.69)	2.51 (3.21)	<0.001
Trail Making - Part A (Time)	50.81 (31.99)	47.53 (15.23)	0.963
Trail Making - Part B (Time)	113.72 (52.32)	110.25 (41.75)	0.986
Logical Memory (WMS-R) - immediate	33.17 (5.51)	26.03 (8.14)	0.004
Logical Memory (WMS-R) - 30'	16.94 (8.53)	4.66 (5.33)	<0.001
Visual Reproduction (WMS-R) - immediate	28.82 (8.18)	22.66 (7.57)	0.023
Visual Reproduction (WMS-R) - 30'	13.64 (11.81)	1.23 (4.32)	<0.001
RAVLT - total	40.08 (8.19)	28.60 (7.81)	<0.001
RAVLT - 30'	7.55 (3.24)	2.31 (1.74)	<0.001
BNT	45.02 (6.85)	42.71 (6.22)	0.138
WCST (Categories)	2.12 (1.54)	1.40 (1.34)	0.280
Verbal Fluency - supermarket	20.81 (3.74)	18.80 (5.82)	0.034
Raven's Colored Matrices	26.90 (4.70)	26.50 (3.50)	0.831

* Mann-Whitney Test; SD, standard deviation; MCI, Mild Cognitive Impairments; WAIS, Wechsler Adult Intelligence Scale; WMS-R, Wechsler Memory Scale - Revised; RAVLT, Rey Auditory Verbal Learning Test; BNT, Boston Naming Test; WCST, Wisconsin Card Sorting Test.

Statistically significant differences were observed between aMCI and AD patients on tasks of constructive and visuoperceptual abilities; attention; language and memory (except for delayed recall of Visual Reproduction test). No statistically significant differences in visual organization (measured by Hooper) and executive functions were noted (measured by WCST) (Table 5).

**Table 5:** Performance of aMCI and AD patients on the neuropsychological tests*.

Tests	Mean±SD		*p*
	Controls	MCI	
Hooper	64.02 (19.75)	55.60 (17.54)	0.041
Block Design (WAIS)	7.10 (3.74)	11.66 (2.50)	<0.001
Rey Figure - Copy	21.39 (10.18)	28.20 (6.60)	0.004
Rey Figure - Memory	0.79 (2.69)	2.51 (3.21)	<0.001
Trail Making - Part A (Time)	92.76 (46.49)	47.53 (15.23)	<0.001
Trail Making - Part B (Time)	215.20 (83.89)	110.25 (41.75)	<0.001
Logical Memory (WMS-R) - immediate	13.78 (8.84)	26.03 (8.14)	<0.001
Logical Memory (WMS-R) - 30'	1.35 (2.93)	4.66 (5.33)	<0.001
Visual Reproduction (WMS-R) - immediate	14.20 (7.33)	22.66 (7.57)	<0.001
Visual Reproduction (WMS-R) - 30'	1.01 (3.95)	1.23 (4.32)	0.863
RAVLT - total	21.00 (7.91)	28.60 (7.81)	<0.001
RAVLT - 30'	1.06 (2.82)	2.31 (1.74)	<0.001
BNT	34.28 (8.93)	42.71 (6.22)	<0.001
WCST (Categories)	0.43 (0.62)	1.40 (1.34)	0.116
Verbal Fluency - supermarket	13.49 (4.74)	18.80 (5.82)	<0.001
Raven's Colored Matrices	17.44 (6.22)	26.50 (3.50)	0.001

* Mann-Whitney Test; SD, standard deviation; MCI, Mild Cognitive Impairments; WAIS, Wechsler Adult Intelligence Scale; WMS-R, Wechsler Memory Scale - Revised; RAVLT, Rey Auditory Verbal Learning Test; BNT, Boston Naming Test; WCST, Wisconsin Card Sorting Test.

## Discussion

Comparison of control group, aMCI and AD patients revealed a statistically significant difference in all tests applied. Our finding of differences among the groups for all cognitive functions evaluated is in line with results of several other studies. Boeve et al.^29^ demonstrated that MCI patients showed intermediate scores on memory tasks which lay between normal subjects and dementia patients. As expected, comparisons between control group and AD patients yielded statistically significant differences in all tests applied, where this result corroborates with characteristics of AD in that beyond memory impairments, other functions including attention, language, reasoning and visuospatial abilities are also damaged.^30^

Neuropsychological assessment was able to discriminate controls from aMCI patients through memory tasks, except for the immediate recall of Visual Reproduction. These findings may be explained since memory begins to decline early in the disease process while the most important evidence for aMCI is poor performance on memory tests^3,4,31-34^. Petersen et al.^1^ compared controls, MCI and mild AD patients by a neuropsychological assessment comprising the MMSE, WAIS-R, WMS-R, Dementia Rating Scale, Free and Cued Selective Reminding Test (FCSRT) and Rey Auditory Verbal Learning Test (RAVLT). The results showed similar performance on measures of general cognition between controls and MCI, but subjects with MCI were more significantly impaired on memory tasks. Perri et al.^35^ investigated different aspects of episodic long-term, short-term and implicit long-term memory in aMCI patients. Results showed normal short-term memory abilities, while the episodic long-term memory showed poorer results in aMCI than in controls. The authors affirmed that although some episodic memory functions were relatively well preserved, others appeared to have deteriorated to a level comparable to that of mild AD patients.

Statistically significant differences were observed between AD and aMCI patients in tasks of constructive and visuoperceptual abilities, attention, language, immediate and delayed recall verbal memory and immediate visual memory. The differences observed in other tasks beyond memory tests can be explained by the fact that AD patients present more severe disorders in many other cognitive domains such as attention, visuospatial abilities, language and complex motor tasks.^1,4,36^ Kramer et al.^37^ compared aMCI subjects, with presumably isolated memory impairment, with controls and very mild AD patients using a battery of neuropsychological tests. As expected, deficits in episodic memory were observed, but the aMCI group performed less well than controls yet better than the AD group on design fluency, category fluency, a set shifting task and the Stroop interference condition. Economou et al.^38^ compared patients with aMCI, mild AD and controls on nonepisodic memory measures. Working memory, processing speed, semantic fluency and complex motor tasks were significantly worse in the mild AD than the aMCI group. Rozzini et al.^39^ investigated the risk factors associated with conversion of aMCI to dementia of Alzheimer type. Subjects who converted to AD over time were classified as demented and subjects that remained unchanged, or became cognitively normal during follow-up were defined as Stable. After a one-year period, demented individuals presented worse performance on phonemic verbal fluency, Raven’s colored matrices, Trail Making test A and B, and in Instrumental Activities of Daily Living than Stable subjects.

No statistically significant difference was observed between AD and aMCI patients in the delayed recall stage of the Visual Reproduction. Alescio-Lautier et al.^40^ found that both MCI and AD patients’ had impairments on visual tasks. Griffith et al.^41^ showed that on the DRS, Initiation/Perseveration scores below 37 and Visual Reproduction Percent Retention scores below 26% correctly identified AD converters with 76.9% sensitivity and 91.7% specificity.

Many studies^4,29,33^ have shown that MCI subjects have a neuropsychological performance which lies between normal older individuals and demented patients. However, neuropsychological features for MCI classification are not yet well established. Studies^42-44^ have suggested that there is probably a continuum between aMCI and AD at early stages, which hampers discrimination among these groups, where the results of our study confirmed these difficulties. According to Nelson and O’Connor^45^ there is no consensus regarding an optimal test battery useful in the detection of MCI. Nonetheless, a well-constructed neuropsychological battery which comprises tests investigating specific aspects of memory complaints can detect subtle cognitive deficits that can easily be overlooked, particularly in patients with a high intellectual baseline. Winblad et al.^2^ suggested that individual slopes of decline in both functional and cognitive performance may be the best measures for diagnosing MCI. According to Celone et al.^46^ a longitudinal follow-up is necessary, however, to determine whether MCI subjects are indeed in the early phases of AD.

The present study confirmed that a well-constructed neuropsychological battery is important to reach differential diagnosis among controls, aMCI and AD patients, but in order to achieve diagnostic accuracy the scales must be used in conjunction with clinical features, functional activities investigation, biomarkers and neuroimaging measures.

In our sample, differences in age and schooling among the groups reached statistical significance. The limitation of this study was the absence of investigation of possible interference of these variables on performance in the neuropsychological tests.
